# Robust sequential working memory recall in heterogeneous cognitive networks

**DOI:** 10.3389/fnsys.2014.00220

**Published:** 2014-11-14

**Authors:** Mikhail I. Rabinovich, Yury Sokolov, Robert Kozma

**Affiliations:** ^1^BioCircuits Institute, University of California San DiegoLa Jolla, CA, USA; ^2^Department of Mathematical Sciences, University of MemphisMemphis, TN, USA

**Keywords:** cognitive dynamics, memory disorders, inhibition, sequential intermittency, complex networks, heteroclinic chimeras

## Abstract

Psychiatric disorders are often caused by partial heterogeneous disinhibition in cognitive networks, controlling sequential and spatial working memory (SWM). Such dynamic connectivity changes suggest that the normal relationship between the neuronal components within the network deteriorates. As a result, competitive network dynamics is qualitatively altered. This dynamics defines the robust recall of the sequential information from memory and, thus, the SWM capacity. To understand pathological and non-pathological bifurcations of the sequential memory dynamics, here we investigate the model of recurrent inhibitory-excitatory networks with heterogeneous inhibition. We consider the ensemble of units with all-to-all inhibitory connections, in which the connection strengths are monotonically distributed at some interval. Based on computer experiments and studying the Lyapunov exponents, we observed and analyzed the new phenomenon—clustered sequential dynamics. The results are interpreted in the context of the winnerless competition principle. Accordingly, clustered sequential dynamics is represented in the phase space of the model by two weakly interacting quasi-attractors. One of them is similar to the sequential heteroclinic chain—the regular image of SWM, while the other is a quasi-chaotic attractor. Coexistence of these quasi-attractors means that the recall of the normal information sequence is intermittently interrupted by episodes with chaotic dynamics. We indicate potential dynamic ways for augmenting damaged working memory and other cognitive functions.

## 1. Introduction

The human brain is a complex net of functionally interconnected regions. Deeper understanding the dynamics of this network is very useful for describing how brain activities transform to task-dependent cognitive processes. This dynamical approach is providing new insights into abnormal brain organization in various psychiatric and neurological disorders. Advances in this area stimulate new discoveries on dynamical disorders related to network connectivity, such as obsessive-compulsive disorder, schizophrenia, dementia, and drug dependence (Chambers et al., 2009; Bystritsky et al., 2012; Hughes et al., 2013). Non-linear dynamical models studying psychopathology must focus on understanding how disturbances in the networks' architecture contribute to cognitive and affective dysfunctions. In particular, it is extremely important to separate emergent dynamics into pathological and non-pathological regimes concerning a specific cognitive task.

As well known, cognitive human resources are finite (Franconeri et al., 2013). When a person becomes sick or its cognitive problem worsens, the performance degrades. There are several possible mechanisms of cognitive resource limitations discuss in the literature; for a review, see Emrich et al. (2013). In this paper, we focus on sequential working memory (SWM) capacity and discuss the instability mechanism related to the length of the information items (chunks) sequence. SWM is a dynamical cognitive network that enables and sustains a set of sequentially ordered mental representations for further recall and processing. The capacity of SWM is quite limited, i.e., it is 5 ± 2 chunks of information at any time instant (Miller, 1956; Bick and Rabinovich, 2009; Rabinovich et al., 2014). The contents of SWM are generally thought to be conscious. The SWM cognitive network, as imaging experiments indicate, consists of several brain modules distributed in different areas of the frontal cortex, sensory cortical regions, hippocampus and some others. These modules interact through the attentional process (Postle, 2006), forming stimulus-dependent spatiotemporal informational modes.

In excitatory-inhibitory cognitive networks that perform SWM, these modes sequentially turn off/ turn on each other according to winnerless competition (WLC) principle. As a result, a stable time-ordered sequence of chunks is formed (Rabinovich et al., 2014). In experiments, the sequence of such switching looks like a chain of metastable states—each state corresponds to the specific mode lasting for a finite time (Stopfer et al., 2003; Jones et al., 2007; Bouchard et al., 2013). The mathematical image of the sequence of metastable states is a stable heteroclinic channel (SHC) in the phase space of the corresponding dynamical model (Afraimovich et al., 2004a; Rabinovich et al., 2012b).

If excitation is constant, cognitive inhibition plays a key role in SWM dynamics and it is the origin of WLC. Cognitive inhibition refers to the mind's ability to tune out stimuli that are irrelevant to the task/process at hand or to the mind's current state (Harnishfeger, 1995; MacLeod, 2007). Cognitive inhibition is caused by several different interacting biological factors. The first is the existence of inhibitory neurotransmitters, or chemicals emitted by brain cells to both communicate and inhibit communication between each other. GABA is an inhibitory transmitter that has been implicated in certain simple behavioral measures of inhibition and the control of behavior in normal and pathological cases; it has been identified in the cerebral cortex (Dempster and Corkill, 1999). Given the cerebral cortex's importance in many brain functions such as memory and thought, the presence of the inhibitory substance GABA supports the cognitive inhibition processes that go on in this area of the brain. Cognitive inhibition is playing a key role in schizophrenia (Westerhausen et al., 2011). The corresponding degradation of the sequence of information items stability is analyzed in this paper. In particular, we are interested in the dynamics of sequential switching in the case of heterogeneity of the SWM network as a result of decreasing cognitive inhibition.

For the description of the modes interaction in excitatory-inhibitory cognitive networks, we use here the traditional model of population dynamics and game theory—generalized Lotka-Volterra equation for N interacting agents (Hofbauer and Sigmund, 1998). In canonic form (see below) this model has N metastable states that are represented in the phase space by saddle fixed points on the axes corresponding to different agent-variables. We consider the case of all-to-all inhibitory connections between participants. Depending on the strengths of the anatomical connections of the subnetworks - motifs or clusters that are embedded in an original larger network can form. These are anatomical motifs. Here we show that in heterogeneous networks one can observe the emergence of dynamical clusters, or dynamical motifs in the phase space, which can be interpreted as temporal unification of different groups of agents.

Cognitive functions, including working memory and attention, involve interconnected networks of brain regions. Recent investigations indicated abnormalities in structural and functional networks in the case of schizophrenia and other disorders, such as depression, obsessive-compulsive disorder, and substance abuse. These conditions are associated with deficits of GABA-mediated synaptic transmission in the brain, when inhibitory connections become weaker in frontal-subcortical neuronal networks (Tekin and Cummings, 2002; Lewis et al., 2005; Murray et al., 2014a).

In contrast with GABA synaptic inhibition in neurophysiological networks, the inhibition in cognition is a concept that is based on behavioral and imaging experiments. In fact, it is a process that has been postulated and modeled by kinetic equations for the competitive cognitive modes (Rabinovich et al., 2012b). In the framework of such models, it is possible to explain changes and deteriorations in the cognitive performance in many domains of psychological and psychiatric research. There are many areas of psychology and cognitive science where the concept of inhibition in global brain networks has been used successfully (Aron, 1982; Constantinidis and Wang, 2004; Gorfein et al., 2007; Joorman et al., 2007; Engelhart et al., 2008; Baumeister et al., 2014; Deco et al., 2014).

Following previous studies, our results indicate potential dynamical ways for augmenting damaged working memory and other cognitive functions. Specifically, we hypothesize that psychiatric and cognitive conditions will express substantial changes in temporal dynamics during key cognitive functions. Furthermore, if these models are successful it would be of great interest to determine if manipulating the organization of the feedback between fMRI time series of working memory (WM) activity through repetitive transcranial stimulation targeting prefrontal cortex can modulate inhibitory WM network and thus provide some control of the chaotic dynamics.

This paper focuses on the analysis of two related cognitive processes, i.e., sequential working memory and attention sharing. The analysis of the dynamics of corresponding functional global networks with inhibitory heterogeneous connections between cognitive modes (information items) revealed a new type of network behavior, which is coined *clustering dynamics*. Clustering dynamics is a sequential activity that includes ordered switching between a few information items, interrupted by intervals with chaotic switching between some other ones. The mathematical image of such intermittent dynamics we named *heteroclinic chimera* as an analog to chimeras observed in networks of phase oscillators (see Omelchenko et al., 2013; Panaggio and Abram, 2014), inspired by neuroscience (Kozma, 1998; Henderson and Robinson, 2011). The observed phenomenon leads to decreasing capacity of the sequential working memory. Moreover, it causes serious impediments in the process of attention sharing among several objects.

## 2. Relation to other modeling studies

In Loh et al. (2007), based on a statistical dynamical model of integrate-and-fire **neuronal** network, the stability of attractor states in prefrontal cortical networks has been analyzed. The authors showed that for the stability of network dynamics is important to have a balance between excitation (NMDA conductance) and inhibition (GABA conductance). In particular, decreasing inhibition reduced the basins of cognitive attractors and destabilized the cognitive task performance that models the schizophrenia symptoms. The concept of excitation-inhibition dynamical balance is supported also by other modeling (Murray et al., 2014b), and experimental studies. In their nice work Murray and coauthors have showed that with constant excitation disinhibition increases random drift and decreases memory precision.

It is important to note that dynamical modeling of sequential neural activities has a long history; for a review, see Rabinovich et al. (2006). In particular, about 30 years ago, two seminal papers have been published (Kleinfeld, 1986; Sompolinsky and Kanter, 1986), in which authors used the same idea, i.e., that sequential patterns were generated by neural networks with time-delayed connections. Recent work about robust sequential memory in networks with controllable steady-state period is related to this idea (Xia et al., 2009). Camperi and Wang (1998) analyzed visual working memory models using networks with cellular bistability. Szatmary and Izhikevich (2010) built a spike-timing network model of working memory using associative short-term synaptic plasticity (STDP). Buonomano (2000) has trained a biophysical network model of decoding temporal information using similar principles. In Seliger et al. (2003), the problem of recalling temporal sequence was solved in the framework of WLC networks. Concluding this obviously incomplete list, we notice that memory storage on short timescales can be maintained by neural activity that passed sequentially through a chain of network states (Goldman, 2009). This mechanism reminds the information propagation along heteroclinic channel in the phase space of WLC network (Rabinovich et al., 2012a).

## 3. Materials and methods

### 3.1. Dynamical principles of cognition: the simplest canonical model

In order to build a non-linear dynamical model of sequential working memory, attention, and other cognitive functions in normal and pathological conditions, we use the following ideas based on brain imaging and behavioral experiments (see Rabinovich et al., 2012b): (i) the equations of the model have to be written for variables that represent the evolution of the temporal coherency of the brain components, and must have solution which correspond to metastable patterns (knowledge) in the brain; (ii) the model must be based on winnerless competitive (WLC) dynamics, a non-linear process of interaction of many informational items or spatiotemporal modes, which guarantees sequential switching between metastable states and potential robustness of transient creativity dynamics; (iii) the model is an open dissipative system where inhibition is balanced by excitation; and (iv) the dynamics of the model has to be sensitive to the changes in memory and environment information.

In our study, we consider a kinetic equation, which can be written as $\dot{x}$_*i*_ = *x*_*i*_ *F*(*x*), where *F*(*x*) is a vector function and *x* = (*x*_1_, …, *x*_*n*_). The Generalized Lotka Volterra (GLV) equation is a specific example of the kinetic equation. Thus, GLV is a non-linear population model with a simple quadratic non-linearity. Moreover, it is known that a system of non-linear equations can be rewritten as system of GLV equations after some suitable transformations (Hernandez-Bermejo et al., 1998). Therefore, the “simple” Lotka Volterra equations can provide a powerful tool for the description of the dynamics of complex networks. Using this approach, one can write the model in the simplest canonical form of Generalized Lotka-Volterra equations (Rabinovich et al., 2006):

(1) $$\tau_{\ell }\frac{dR_{\ell }}{dt}=R_{\ell }\left(\gamma_{\ell }-\sum_{k\;=\;1}^{N}a_{\ell ,k}R_{k}\right),$$

where *R*_*ℓ*_ is the level of activity of *ℓ*-th mode, *ℓ* = 1, …, *N*. Information mode variable *R*_*ℓ*_ must be positive or equal to zero for all *ℓ*. *N* is the total number of modes describing the components that interact to perform a specific cognitive task. Time constants τ_*ℓ*_ are fixed for a given system *ℓ*, while parameters *a*_*ℓ*, *k*_ describe the inhibitory connections between mode *ℓ* and *k*, while *a*_*ℓ*, *ℓ*_ = 1 for any *ℓ*, and γ_*ℓ*_ is the strength of the stimulation of mode *ℓ*. It is important that, in general, the elements of this matrix are controlled by cognitive tasks.

The dynamics of the cognitive network is extremely sensitive to the diversity of inhibitory connections. In this work, we model cognitive diseases through heterogeneous decrease of inhibitory activity in the cortex.

It is very important to emphasize that the complexity of the corresponding model is determined by the numbers of variables (agents) and the task dependent functional hierarchical architecture of the cognitive networks (Rabinovich et al., 2014). The individual dynamics of the agents is of secondary importance and it can be selected based on a suitably simple model. While numerous models exist that explain various aspects of cortical behavior, even simple, parsimonious GLV models (Bick and Rabinovich, 2009; Rabinovich et al., 2010) can produce intermittent chaotic behavior by a change in inhibitory weights, which provide insights into dynamical behaviors that may mirror pathological conditions.

### 3.2. Lotka-volterra networks. gallery of the phenomena

GLV model is very important and popular model for the analysis of multi agent non-equilibrium dynamics in ecology, biochemistry, and neuroscience. There is a huge amount of publications about GLV dynamics. Here we recall the main phenomena that are described by this model, which have been observed over a wide range of the control parameters.

**Figure 1:**
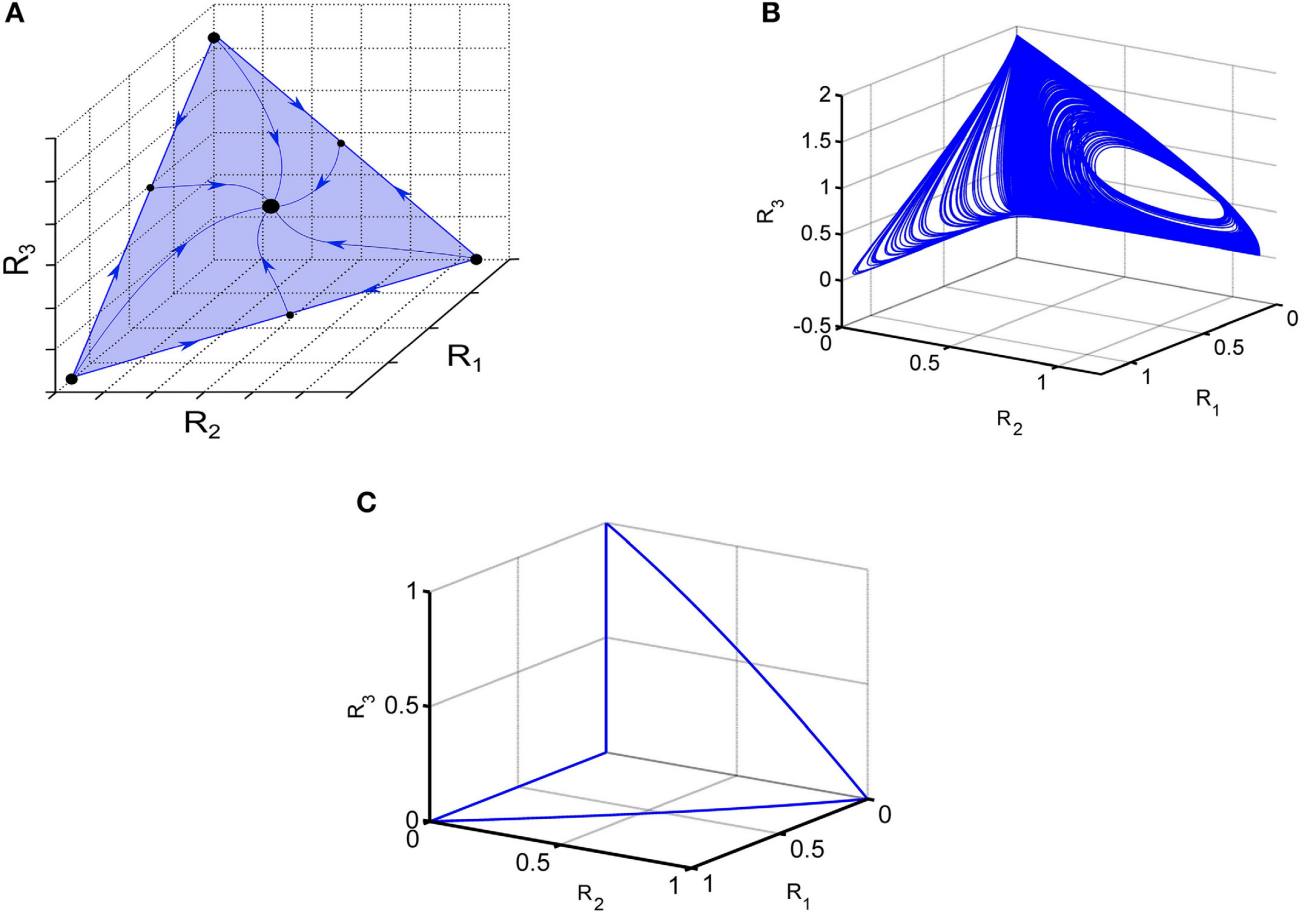
**Illustration of various dynamical regimes of the Lotka-Volterra system**. **(A)** Example of a system when all trajectories converge to one stable fixed point, illustrating the coexistence of 3 modes. **(B)** Portrait of irregular switching in a 6-dimensional inhibitory LV network. 3D projection of the phase portrait of the observed strange attractor Equation (1). **(C)** Winnerless competitive dynamics of a network with random non-symmetric connections; 3D projection is shown; see Huerta and Rabinovich (2004).

If the connection matrix is symmetric the GLV model is a gradient system and demonstrates monotonic dissipative dynamics (Hirsch and Smith, 2005). In particular, one can observe multistability like in associative memory neural networks (Hopfeld, 1982; Cohen and Grossberg, 1983; Yi et al., 2003). In the case of moderate inhibition, the typical regime is winner-share-all (Fukai and Tanaka, 1997). Phase portrait of such stable regime of the symmetric high-dimensional model Equation (1) is shown in Figure 1A.

If the connection matrix is non-symmetric, the GLV model dynamics is extremely rich. For *N* > 3 the dynamics of GLV system can be chaotic (Arneodo et al., 1982; Takeuchi, 1996; Varona et al., 2002; Rabinovich et al., 2012b). An example of chaotic behavior in the GLV model with 6-modes is shown in Figure 1B. Another example is the transient dynamics representing cognitive information processing such as attention switching or sequential working memory stability (Bick and Rabinovich, 2009; Rabinovich et al., 2014); stable heteroclinic transients are illustrated in Figure 1C.

The key issue for the present study is understanding the origin of the SHC instability in the framework of model Equation (1) with heterogeneous connections. The results are important for the description of sequential information processing; for a review, see Rabinovich et al. (2012a).

### 3.3. Stability of the information sequence. saddle values

Robust transient dynamics is organized in the phase space typically as a chain of sequentially switching of metastable states—saddle points. The mathematical image of such dynamics is a stable heteroclinic channel—the vicinity of the chain of saddles coupled sequentially by their unstable separatrices. The chain can be finite, i.e., ending at the simple attractor (stable fixed point), or it can be periodic, asymptotically reaching a heteroclinic cycle.

Let *A*_*i*_ = (0, …, 0, γ_*i*_, 0, …, 0) be an equilibrium point of the system Equation (1), *i* = 1, …, *N*. If λ^(*i*)^_1_, …, λ^(*i*)^_*N*_ are eigenvalues of the matrix of the system linearized at *A*_*i*_, that are ordered as follows λ^(*i*)^_1_ > … ≥ Re λ^(*i*)^_*k*_*i*__ > 0 > Re λ^(*i*)^_*k*_*i*_ +1_ ≥ … ≥ Re λ^(*i*)^_*N*_ then *A*_*i*_ is a saddle with *k*_*i*_-dimensional unstable manifold.

When the unstable manifolds of the saddles are one-dimensional, i.e., *k*_*i*_ = 1 for all *i*, the stability of a SHC depends on the ratios of the compression of the phase volume to the stretching of it in the vicinity of the channel. These ratios are called saddle values and they can be defined as ν_*i*_ = − Re λ^(*i*)^_2_/λ^(*i*)^_1_. Thus, if ν_*i*_ > 1, the saddle is called dissipative and the trajectories get closer to the unstable manifold of the saddle after passing through its neighborhood. The mechanism of the SHC emergence in dissipative systems is the Winnerless Competition that can guarantee the sequential switching of agents activity in networks with non-symmetric inhibitory coupling (Rabinovich et al., 2006, 2012b; Bick and Rabinovich, 2010).

The conditions of the existence and stability of the heteroclinic contour with constant uniform stimulation strength γ_*i*_ = 1 for any *i* are given in Afraimovich et al. (2004a). The conditions of existence and stability of the heteroclinic sequence with different values of γ_*i*_ were obtained in Afraimovich et al. (2004b). To support the proposed interpretation of cognitive dynamics using heteroclinic chimera, we provide detailed mathematical analysis in the Appendix.

## 4. Results

### 4.1. Dependence of the network dynamics on the distribution of inhibitory connections strengths

**Figure 2:**
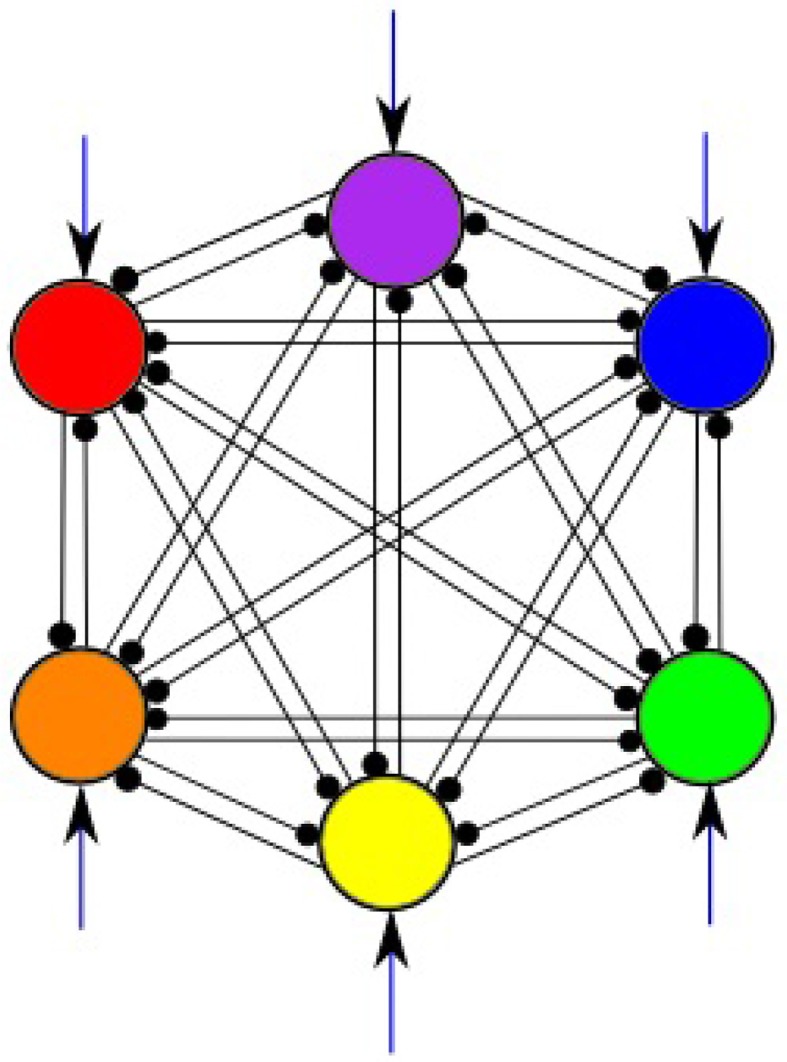
**All-to-all inhibitory interactions between 6 modes (information items) of the cognitive network; excitatory self connections are not shown**. Black arrows represent incoming signals.

In this work, a system of 6 LV Equations (1) is studied. Figure 2 illustrates the general structure of such system with all-to-all connections among 6 modes shown in 6 different colors; external inputs are marked by arrows pointing to each mode. We are able to select the parameters of the model to guarantee the regime of regular sequential working memory. In order to produce a system having robust heteroclinic contour, the inhibitory connections are chosen in two groups, i.e., with weights greater than one and smaller than one, respectively, while the self-inhibition weights are equal to one. Moreover, the weights in each of these subsets have a limited spread, i.e., they are concentrated around a particular value. Also, the strength of the inhibition is growing when the number of interacting modes increases; for details, see Bick and Rabinovich (2010). In the pathological case of weakened inhibition, on the other hand, we may expect that the strength of some inhibitory connections to approach zero.

### 4.2. Dynamical clustering in heterogeneous networks. intermittent sequences and heteroclinic chimera

**Figure 3:**
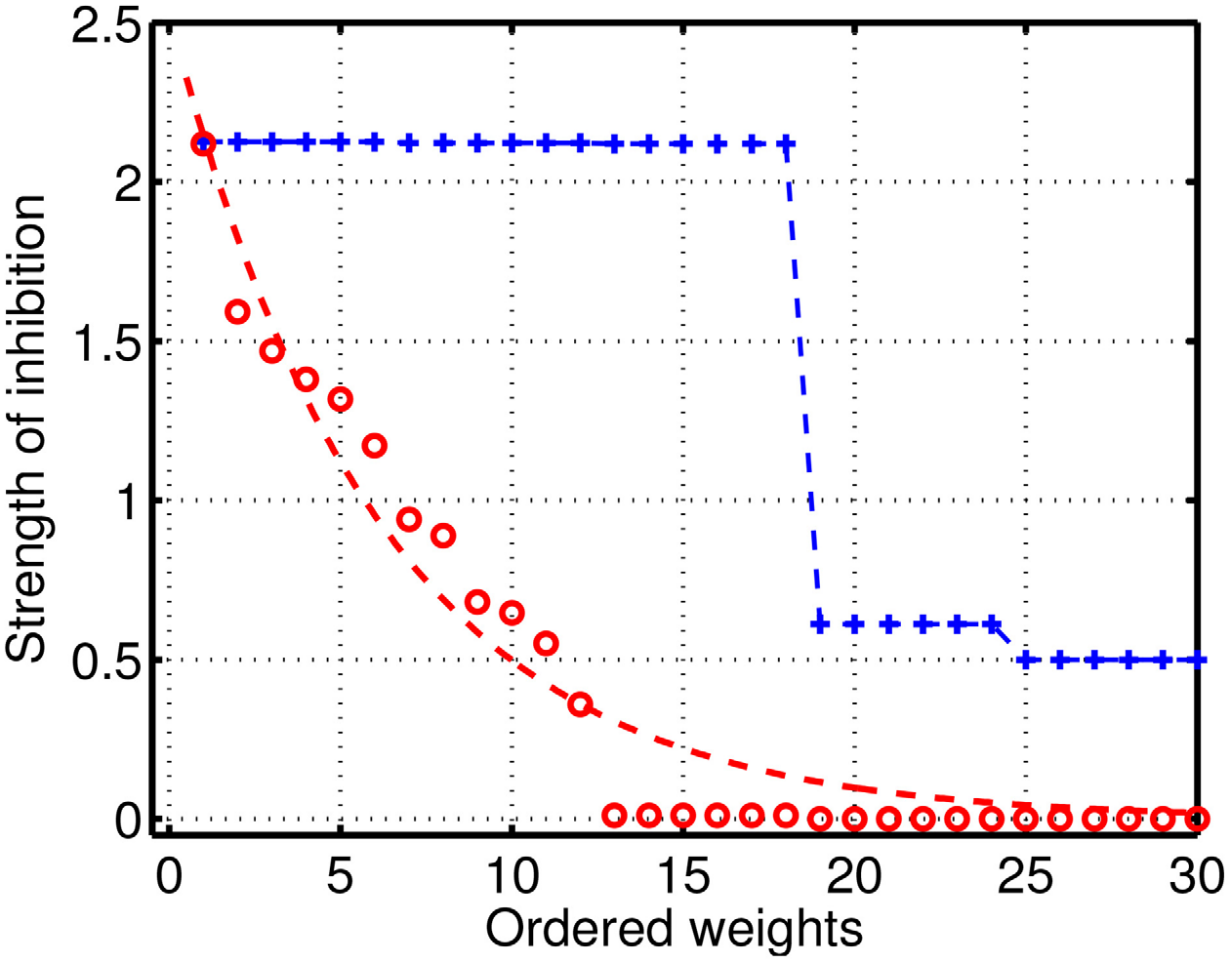
**Distribution of the strengths of the inhibitory connections in the GLV network with 6 participants**. Blue: connectivity in a network producing normal SHC behavior; the large weights represent two triangle motifs with strong constant connectivity values, while there is a group of small weights describing weak connectivity between the motifs. Red: illustrates the pathological case with significantly reduced inhibitory connections with respect to normal SHC case, which are distributed over the parameter range; the dotted red line is a simple exponential fit shown for better visibility.

To analyze in detail the case of reduced/intermediate strength of coupling when quasi-periodic heteroclinic dynamics and chaos co-exist in a mutually coupled system, we performed extensive simulations with various sets of parameters. Examples of the distribution of the inhibitory weights in the GLV system with 6 modes are given in Figure 3. Blue color illustrates connectivity in a network producing normal SHC behavior, when the strong weights correspond to two triangle motifs, while there is a group of small weights describing weak connectivity between the motifs. The degraded (pathological) case is shown in red and it has significantly reduced inhibitory connection values with respect to the normal SHC case. The magnitudes of the weights are distributed over a range of parameters; the dotted red line illustrates a simple exponential fit for better visibility.

**Figure 4:**
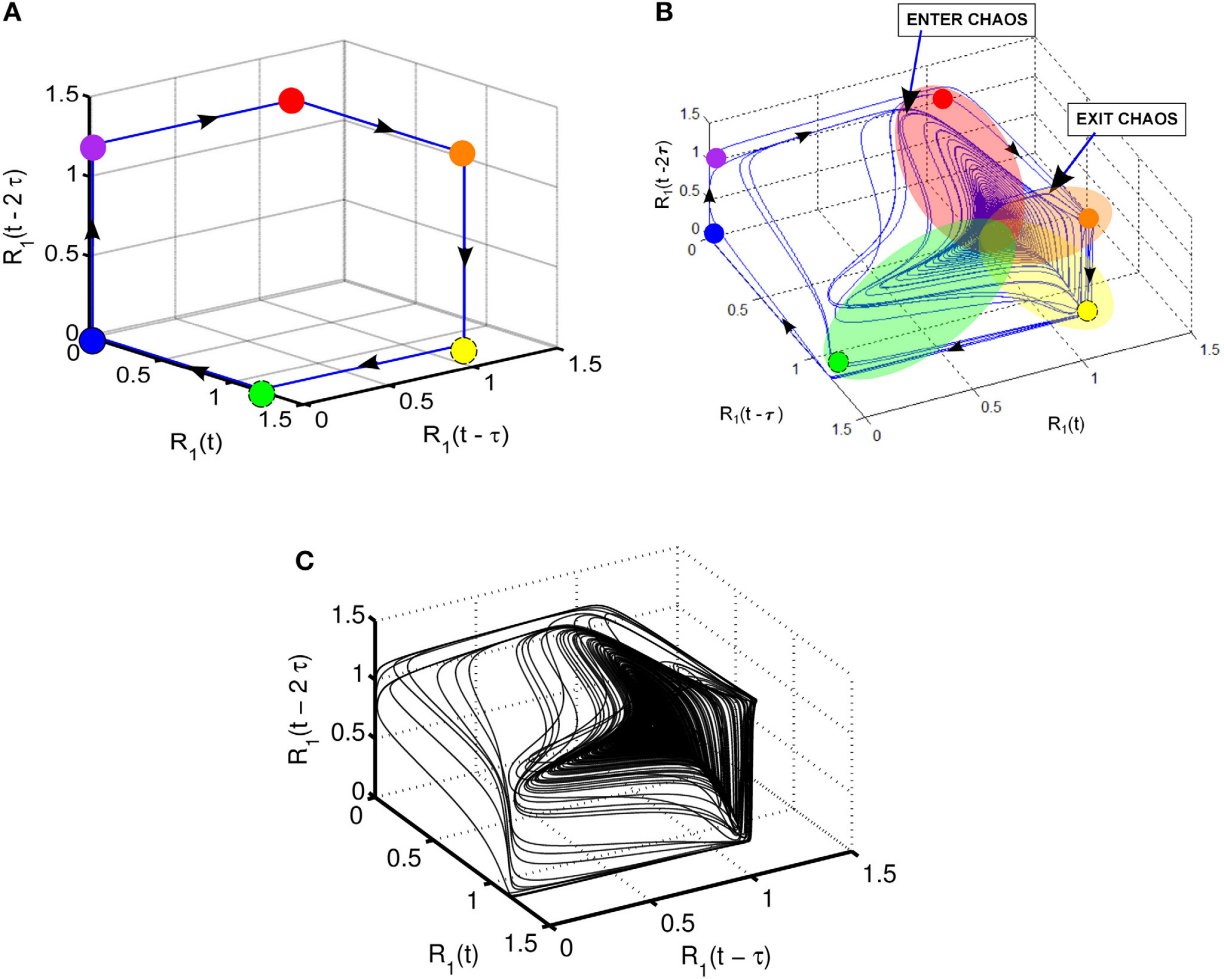
**Illustration of various dynamic regimes using 3-dimensional phase portrait with time-lagged reconstruction of *R*_1_; (A) normal regime with stable heteroclinic channel (SHC); (B) abnormal regime: coexistence of SHC and quasi-chaotic attractor—heteroclinic chimera; (C) abnormal regime: coexisting SHC and quasi-chaotic attractor for longer time series with up to 10^5^ points**.

Takens' theorem (Takens, 1981) can be used to reconstruct high-dimensional attractors from the time series of a variable using time-delayed coordinate embedding. Note that time delay τ can be selected according to the given problem to produce a suitable display of the phase portrait. For example, *R*_1_(*t*) and its time-lagged copies *R*_1_(*t* − τ) and *R*_1_(*t* − 2 τ) are used in Figure 4 to show the 3-dimensional phase portrait with time-lagged reconstruction. The case of τ = 150 is used in this display; the direction of the trajectory is illustrated by arrows. Figure 4A shows the case of SHC corresponding to normal parameters. On the other hand, the attractor produced by reduced strength of coupling parameters given in Figure 4 reflecting pathological conditions is shown in Figure 4B. Here a highly complex dynamics emerges resembling the Roessler strange attractor with two wings (Kennel et al., 1992). Specifically, the attractor in Figure 4B has a main wing and a secondary wing. The main wing is shown by overlapping red, orange, yellow, and green patches. There is a second wing, which is less pronounced and approaches but does not quite reach the blue and magenta dots. The secondary wing starts when the trajectories exit the main wing shown as *Exit* in Figure 4B, and later return to the main wing through the region shown as *Enter*. Figure 4C depicts a more detailed view of the two-wing attractor using more detailed computer simulations with about 10^5^ time steps.

Quantitative evaluation of the Lyapunov exponents confirms the coexistence of heteroclinic cycles and chaos. Namely, we have two positive Lyapunov exponents, one small negative value close to zero, one small negative exponent, and two large negative exponents. The exact Lyapunov exponent values corresponding to parameters shown in Figure 3 are as follows: λ_1_ = 0.0061± 0.0005, λ_2_ = 0.0008± 0.0001, λ_3_ = −0.0019 ± 0.0015, λ_4_ = −0.0127± 0.0019, λ_5_ = −0.6654 ± 0.0004, λ_6_ = −1.4409± 0.0002. We explored a variety of systems close and further away from the heteroclinic cycles. The above conclusions have been confirmed, i.e., we have two positive Lyapunov exponents, one close to zero, and the rest are negative. Our results show that two different dynamic regimes coexist in a single system of coupled agents with non-oscillatory intrinsic dynamics, similarly to the chimera states described recently in the literature (Abrams and Strogatz, 2004; Hagerstrom et al., 2012; Omelchenko et al., 2012). Earlier manifestations of chimera states have been in ensembles of phase oscillators. In our case of WLC, however, we observe amplitude clusterization.

Dynamical clustering means the separation of the phase flow into several qualitatively different components. In our example we have two components, one includes quasi-heteroclinic regular trajectories, while the other is a transient chaotic set. We named such complex image as **heteroclinic chimera**. We observe that the network's cooperative dynamics is dominated by the weak interaction between just a few (in our case two) dynamical sub-networks, although the monotonic heterogeneity of the connection strengths between agents would allow more distributed interaction dynamics.

We interpret this behavior as emergent granulation of the distributed dynamics into the interaction of just a few sub-networks. These sub-networks could be formed through the collaboration between agents *R*_*i*_, *i* = 1, …, 6 with the strongest mutual interaction between each others. We have tested this interpretation in the case of weak interaction between two strongly interconnected sub-networks, each with 3 modes (triangles). Our results indicate that in the case of coupled triangles we have topologically the same phase portrait of heteroclinic chimera as we observed in the original system with distributed parameters. We pointed out earlier, following Equation 1, that information mode variable *R*_*i*_ is either positive or zero, which determines the differences between the previously known phase chimera characterizing the dynamics of the network of phase oscillators, and the new heteroclinic chimera observed here in the case of the WLC processes. These results point to the importance of conducting a rigorous analysis in the case of weak inhibitory connections; details are given in the Appendix.

**Figure 5:**
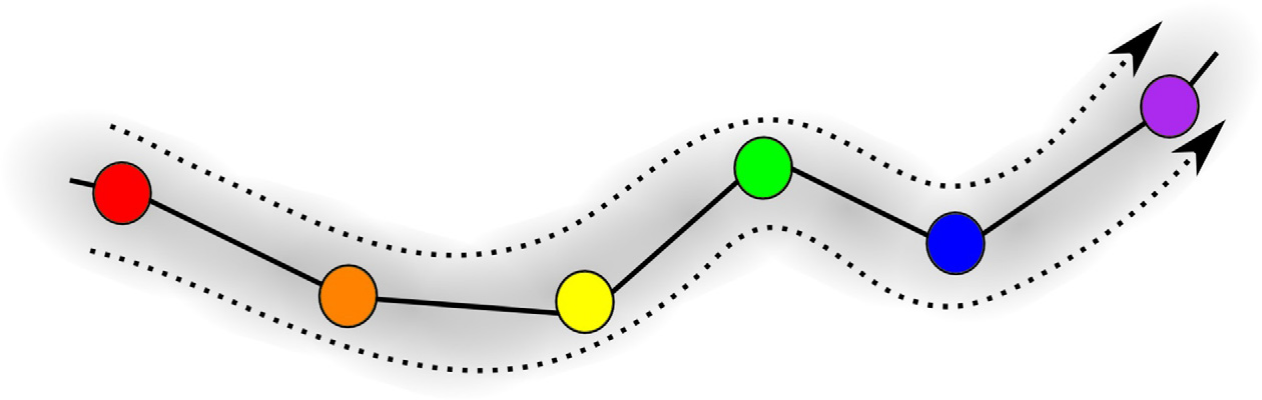
**Illustration of the trajectory in an SHC progressing through the sequence of saddle points; different memory patterns are shown in different colors, i.e., red, orange, yellow, green, blue, and violet**.

**Figure 6:**
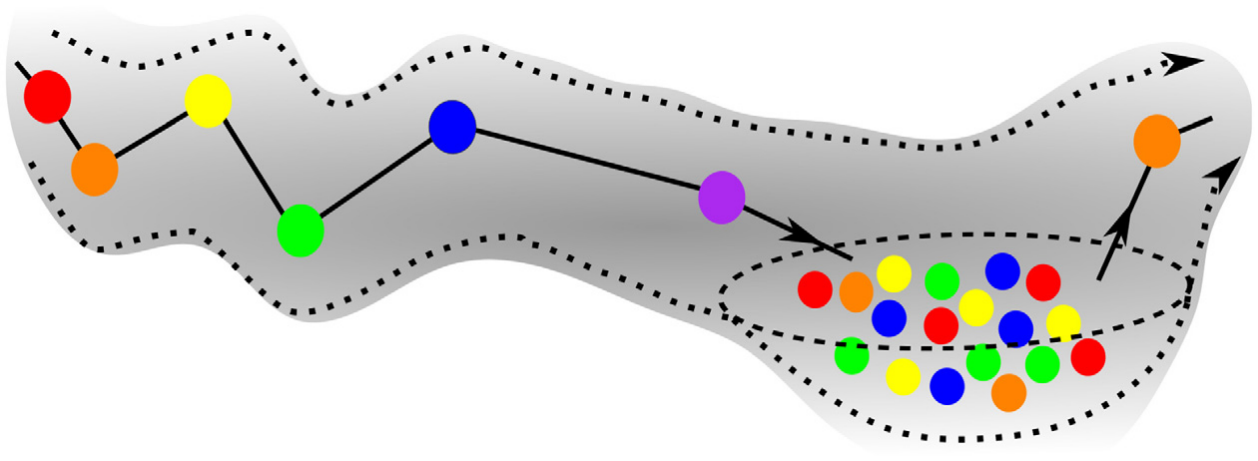
**Schematics of the trajectory of a pathological case of GLV equations, when the initial sequence enters a chaotic valley after the violet unit**. The system stays in the valley for some time, but ultimately escapes it and resumes the regular SHC switching, see exit at the orange unit.

Figure 5 is the schematic image of a SHC modeling normal cognitive functions with sequential switching between various memory items. The consecutive patterns are symbolized by circles of different colors for simplicity. The appearance of the transient chaotic dynamics is illustrated in Figure 6, when the sequence of the patterns enters a *valley*, in which various colors are mixed in a chaotic fashion. This is the model of abnormal cognitive dynamics when the regular sequential memory dynamics breaks down. After some period of time, however, the trajectory leaves the chaotic *valley*, e.g., through the orange unit, and it resumes the regular sequential switching pattern.

**Figure 7:**
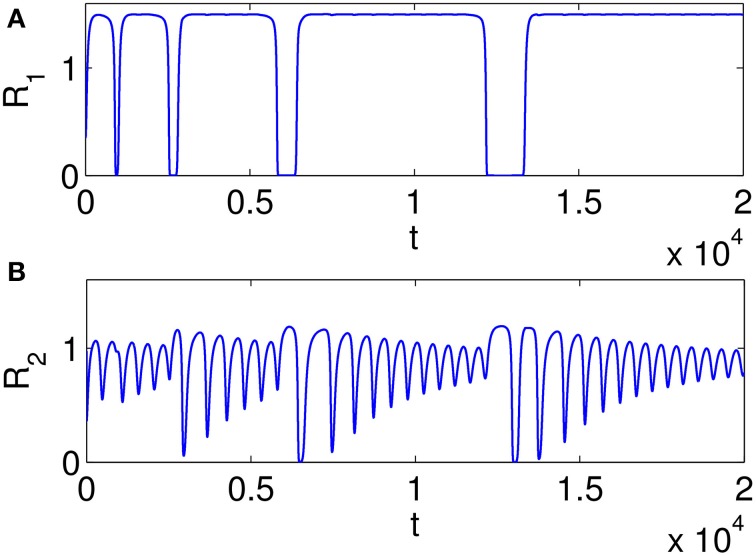
**Examples of time series illustrating heteroclinic chimera in a GLV network with degraded inhibitor weights; the GLV network has 6 units, and two units are shown here. (A)** Variable *R*_1_(*t*) exhibits SHC regime with stable values over extended time periods; **(B)** Variable *R*_2_(*t*) has frequent irregular oscillations; the determined positive Lyapunov exponent indicates the existence of chaos.

**Figure 8:**
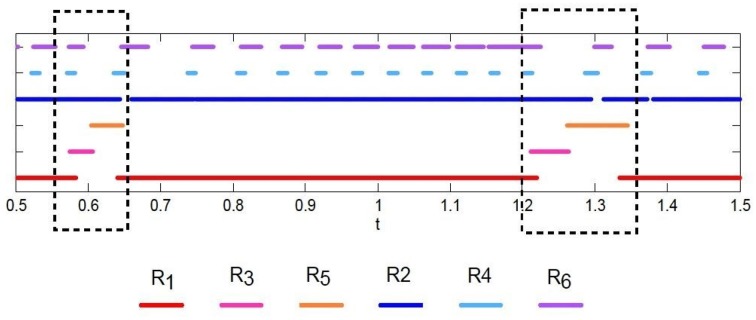
**Raster plot of intermittent sequences of the variables of the GLV system in the case of heteroclinic chimera dynamics; the 6 variables are shown as *R*_1_, *R*_2_, *R*_3_, *R*_4_, *R*_5_, and *R*_6_, respectively**. A given variable is shown in the plot if its amplitude exceeds a threshold value; the threshold used here is 0.1. Note the preservation of sequential switching in the boxes marked by dashed lines.

Figure 7 illustrates heteroclinic chimera in a GLV network with degraded inhibitor weights. This GLV network has 6 units, from which two units are shown here, *R*_1_ and *R*_2_, respectively. Figure 7A depicts variable *R*_1_(t), which exhibits SHC regime with stable values over extended time periods. Figure 7B shows the temporal evolution of variable *R*_2_(t), which has frequent irregular oscillations. The determined positive Lyapunov exponent indicates the existence of chaos. The dynamics of all 6 variables *R*_*i*_, *i* = 1, …, 6 of the abnormal system is summarized in Figure 8 using a raster plot. A given variable is shown in the plot if its amplitude exceeds a threshold value (of 0.1). Note the preservation of sequential switching in the boxes marked by dashed lines. While some of the variables apparently maintain stable heteroclinic trajectories, others exhibit intermittent oscillations and chaos, as a manifestation of heteroclinic chimera dynamics.

## 5. Discussion

This paper analyzes the role of the heterogeneity of inhibitory connections in a cognitive network that models cognitive sequential information processing. The introduced model has been applied for the description and prediction of many cognitive processes like working memory, attention, and decision making. Here we suggested a plausible dynamical mechanism to study the deterioration of the working memory. Sequential order has been destroyed as the result of pathological heterogeneous decrease of some of the inhibitory connections. This mechanism is related to a new dynamical phenomenon: dynamical clustering of information items (cognitive modes) in networks with heterogeneous inhibitory connections.

The corresponding phenomenon is coined here “heteroclinic chimera.” In the case of heteroclinic chimera, we observe the coexistence of chaotic and heteroclinic cycle behavior, thus the chimera property is expressed through sequential amplitude coordination. An important distinction between the previously described phase chimera and heteroclinic chimera is that phase chimera represents phase dynamics and it does not relate to the temporal sequence of items. The phenomenon of dynamical clustering described in this work in the case of heterogeneous inhibitory connections in model Equation 1 is robust. In Sokolov et al. (2014) the dynamics of the model is studied in the presence of multiplicative noise. It is shown that the noise does not change the qualitative picture of the dynamics.

Studies focusing on the analysis of sequential non-linear brain dynamics in the case of psychiatric disorders attract the interest of medical doctors. Results exist related to anxiety and depression (Bystritsky et al., 2014), and to obsessive-compulsive disorder (Bystritsky et al., 2012; Schiepek et al., 2013). Some psychiatrists anticipate that analysis of non-linear sequential dynamics will lead to changes in cognitive behavioral therapies (Kronemyer and Bystritsky, 2014).

There are several fMRI experiments that focused on the spatiotemporal analysis of the representation of informational items in short-time memory (Attout et al., 2014; D'Argembeau et al., 2014, and references therein). In addition, some experiments show the disruption of functional cortical networks in the case of psychiatric disorders including schizophrenia (Baker et al., 2014; Bittner et al., 2014). However, no comprehensive analysis have been completed yet concerning the spatiotemporal dynamics of functional cortical networks, in the case of patients with schizophrenia who do not recall ordered information from SWM. Together with our colleague Alan Simmons (UCSD Dept. of Psychiatry), we plan to conduct such analyses and compare the results with our predictions. In particular, we intend to measure the voxels in functional time series in pathological conditions, i.e., signal intensity vs. time activity in parietal and prefrontal cortexes, to characterize the performance of SWM recall. The modeling results are represented by the *R*_*i*_(*t*) time series. The mathematical image of the damaged SWM with heteroclinic chimera is in fact an intermittent chaotic dynamical regime. There are several successful methods for controlling chaotic dynamics; see, for example Sieber et al. (2014), which is a successful approach to modulate the irregular activities by feedback.

Observations in neuroimaging studies were used to describe the neural correlates of cognitive deficits in attention, working memory and executive functions in patients with Huntington's disease (Montoya et al., 2006). The chaotic behavior of clustered sequential dynamics can serve as a model of Huntington's Chorea.

A related important cognitive problem involves abnormalities in attention switching and focusing, which can be described by the proposed dynamical model; see also Rabinovich et al. (2013). Of the many clinical features of schizophrenia, disturbances in certain cognitive processes, such as impairments in attention, memory and executive functions (that is, the ability to plan, initiate, and regulate goal directed behavior), might represent the core features of the illness (Elvevag and Goldberg, 2000). There is increasing evidence indicating that such disorders are related to decreasing level of inhibition in cortical inhibitory circuits (Lewis et al., 2005). In recent studies, schizophrenia patients has been tested to answer the question: are they impaired relative to controls in sustaining attention, switching attention, or both (Smid et al., 2013). The results supported the hypothesis that schizophrenia is associated with attention switching, while the mechanisms of sustained attention remains largely intact. Our results give a dynamical interpretation to these observations.

If the GLV model suggested is successful in characterizing the differences in temporal dynamics in normal and pathological samples then it would be of great interest in determining if this could guide a potential intervention in these dynamics to either mimic or ameliorate the core symptomologies of these pathologies. We expect that it would be very promising to provide feedback based on functional sequential cortical activity during memory recall, i.e., the time series representing sequential switching between metastable states (Polyn et al., 2005; Norman et al., 2006), by repetitive transcranial brain stimulation. This feedback may involve either repetitive magnetic stimulation (Barr et al., 2013) or ultrasound stimulation (Hameroff et al., 2013; Mueller et al., 2014). Such feedback has to support the correct sequential switching between corresponding recalled information items from SWM.

SHC is the mathematical image of regular sequential switching of attention as it is postulated in Rabinovich et al. (2013). Regular sequential switching between attention modalities is maintained in our model at normal conditions. In the case of the pathological inhibitory strength distribution (selective decrease of inhibitory weights), the regular sequential switching of attention focus is impaired. As a result, the sequential switching is intermittently interrupted by periods of irregular/chaotic dynamics, and the attention switching process becomes uncontrolled. Our modeling results are also in agreement with recent works (Colzato et al., 2007; Tomasi et al., 2010) showing that chronic cocaine use is associated with disrupted inhibitory connections in the brain. In particular, findings in Tomasi et al. (2010) suggest that decreased functional dopaminergic inhibitory connectivity of the midbrain interferes with the activation and deactivation signals associated with sustained attention in cocaine addicts.

## Author contributions

All co-authors have equal contribution to all steps of preparation of this article and they approved the version to be published.

### Conflict of interest statement

The authors declare that the research was conducted in the absence of any commercial or financial relationships that could be construed as a potential conflict of interest.

## Appendix

Here the case of GLV with weak inhibitory connections are studied. For convenience, let us introduce the notation for foregoing sub-networks *S*_1_ and *S*_2_, respectively. Both subsystems have 3 modes, *S*_1_ contains modes *R*_1_, *R*_3_, and *R*_5_, while *S*_2_ includes *R*_2_, *R*_4_, and *R*_6_. We consider the case of weak, uniform connections between the two subsystems. Accordingly, κ_1_ and κ_2_ denote the connection strength from *S*_1_ to *S*_2_ and from *S*_2_ to *S*_1_, respectively. For the sake of simplicity, here we assume κ_2_ = 0 and we study bifurcation only with respect to one parameter κ = κ_1_. Note that the results of this section are readily extendable to the case κ_2_ ≠ 0.

Based on the definition of subsystems *S*_1_ and *S*_2_, clearly two independent stable heteroclinic cycles exist for κ_1_ = 0. Further, it is expected that the two heteroclinic cycles are maintained for very weak coupling 0 < κ < < 1. Increasing κ in the small to intermediate coupling levels, complex dynamics may emerge, such as simultaneous presence of chaos and heteroclinic cycles. The upper bound of this region is marked as κ^*^. Finally, the cycles must collapse if coupling exceeds a high threshold value (κ > > 1).

We start with the definition of several quantities, which allow us to separate the whole domain of coupling parameter κ into regions with different types of behavior. The threshold values are expressed as follows: κ^*^ = *max*_*i*_ {γ_*i* + 1_ γ_*i*_}, *i* = 1, …, 6, where γ_*i*_ is the strength of stimulation of mode *i* (see Equation 1). Further, let us characterize each equilibria. In the following considerations, all indices are written with respect to (mod 6) and we use of the following convention {*i* ∈ {0, …, *n*} | *i* = *n* ( mod *n*)} = *n*. Let us define for each *i* = 1, …, 6 the corresponding set of two numbers *i* = {(*i* ± 2) mod 6}.

Further notations are: *k*^*^_*odd*_ = max{*k*_1_, *k*_3_, *k*_5_} and *k*^*^_*even*_ = max{*k*_2_, *k*_4_, *k*_6_}, and indexes defined as *i*^*^_*odd*_ = arg max{*k*^*^_*odd*_} and *i*^*^_*even*_ = arg max{*k*^*^_*even*_}, where *k*_*i*_ is defined by

(2) $$k_{i}=\frac{\gamma_{\left(i\;+\;1\right)}(-\sum_{k\in \bar{i}}\gamma_{k}a_{ki}+\gamma_{i}\prod_{k\in \bar{i}}a_{ki})}{\prod_{k\in \bar{i}}\left(\gamma_{k}-\gamma_{i}a_{ki}\right)}$$

In the following considerations, we use quantities *k*' = max{*k*_*i*^*^_*odd*_ − 2_, *k*_*i*^*^_*odd*_ + 2_} and *k*″ = max{*k*_*i*^*^_*even*_ − 2_, *k*_*i*^*^_*even*_ + 2_}. It is easy to see that *k*' and *k*″ are larger than the other thresholds. In the following description, we assume that *k*′ < *k*″ unless it is specified otherwise.

There exists a value of coupling parameter 0 < *k*^0^ < < 1 such that for κ∈(0, *k*^0^) the coupled system exhibits two heteroclinic cycles. For κ ∈ (*k*^*o*^, κ^*^) various complex behaviors emerge, with the possibility of co-existing chaotic attractor and heteroclinic cycle. In the case of κ ∈ (κ^*^, *k*′) the system converges to a fixed point. For κ ∈ (*k*′, *k*″), a heteroclinic cycle in one system coexists with zero fixed points in the other system. Finally, for κ > *k*″ the coupled system collapses. Thus, we have the following regions

(3) $$0<k^{o}<\kappa^{*}<k^{\prime }\leq k^{\prime \prime }<\infty .$$

If *k*′ = *k*″ then the conclusion still holds with the difference that the behavior corresponding to values between *k*' and *k*" does not occur. The present results were derived based on the dissipative property of the saddle point. In other words, the equilibrium point attracts trajectories in its neighborhood if it is dissipative point. However, if the dissipative property of the saddle point changes, i.e., the saddle value is no more greater than one due to the increase of κ, we may observe that the orbits move in directions away from equilibria. For this reason, when the coupling parameter is large (κ > *k*′), the origin will attract the trajectories of one of the subsystems. Under this scenario, we have one subsystem (i.e., *S*1 or *S*2) embedded in 6 dimension, and this subsystem behaves as in the case κ = 0. In other words, considering the phase space ℝ^6^ = ℝ^3^ ⊕ ℝ^3^, if one subsystem vanishes we deal with the subspace where all coordinates of this subsystem are zero, so the other subsystem behaves like in the case κ = 0.

In the case of κ ∈ (κ^*^, *k*′), the central eigenspace of each equilibria stays the same. However, the number of stable non-leading eigenvalues is increased to the maximum possible value, thus fixed points appear. In the most challenging case when κ ∈ (*k*^0^, κ^*^), the equilibria which form the heteroclinic cycles have different number of stable and unstable non-leading eigenvalues. This case is possible due to the non-symmetry of the equations, which describe the coupled system with mutual inhibition between its subsystems. To put it simply, there is no symmetry group between its subsystems coupling.
